# Clinical Development of Immune Checkpoint Inhibitors

**DOI:** 10.1155/2015/605478

**Published:** 2015-06-16

**Authors:** Ayumu Ito, Shunsuke Kondo, Kohei Tada, Shigehisa Kitano

**Affiliations:** ^1^Department of Hematopoietic Stem Cell Transplantation, National Cancer Center Hospital, 5-1-1 Tsukiji, Chuo-ku, Tokyo 104-0045, Japan; ^2^Department of Experimental Therapeutics, Exploratory Oncology Research and Clinical Trial Center (EPOC), National Cancer Center, 5-1-1 Tsukiji, Chuo-ku, Tokyo 104-0045, Japan; ^3^Department of Hepatobiliary and Pancreatic Oncology, National Cancer Center Hospital, 5-1-1 Tsukiji, Chuo-ku, Tokyo 104-0045, Japan; ^4^Division of Cancer Immunotherapy, Exploratory Oncology Research and Clinical Trial Center (EPOC), National Cancer Center, 5-1-1 Tsukiji, Chuo-ku, Tokyo 104-0045, Japan

## Abstract

Recent progress in cancer immunotherapy has been remarkable. Most striking are the clinical development and approval of immunomodulators, also known as immune checkpoint inhibitors. These monoclonal antibodies (mAb) are directed to immune checkpoint molecules, which are expressed on immune cells and mediate signals to attenuate excessive immune reactions. Although mAbs targeting tumor associated antigens, such as anti-CD20 mAb and anti-Her2 mAb, directly recognize tumor cells and induce cell death, immune checkpoint inhibitors restore and augment the antitumor immune activities of cytotoxic T cells by blocking immune checkpoint molecules on T cells or their ligands on antigen presenting and tumor cells. Based on preclinical data, many clinical trials have demonstrated the acceptable safety profiles and efficacies of immune checkpoint inhibitors in a variety of cancers. The first in class approved immune checkpoint inhibitor is ipilimumab, an anti-CTLA-4 (cytotoxic T lymphocyte antigen-4) mAb. Two pivotal phase III randomized controlled trials demonstrated a survival benefit in patients with metastatic melanoma. In 2011, the US Food and Drug Administration (FDA) approved ipilimumab for metastatic melanoma. Several clinical trials have since investigated new agents, alone and in combination, for various cancers. In this review, we discuss the current development status of and future challenges in utilizing immune checkpoint inhibitors.

## 1. Introduction

In this decade, remarkable progress has been made in the clinical application of cancer immunotherapies. Most notable is the emergence of immune checkpoint inhibitors. Large-scale clinical trials have shown their feasibility and efficacy for patients with advanced malignancies. The therapeutic targets, or “immune checkpoints,” are also known as coinhibitory molecules or costimulatory molecules expressed on T cells.

As the name implies, costimulatory/inhibitory molecules mediate positive/negative signals that modify MHC-TCR (major histocompatibility complex-T-cell receptor) signaling pathways. These signals each regulate T-cell survival, proliferation, differentiation, or responsiveness to cognate antigens. The net effect depends on the balance among signals [1]. T-cell activation requires costimulatory signals. If they contact antigens without costimulatory ligands on antigen presenting cells (APCs), T cells remain inactivated in a state of anergy.

Coinhibitory molecules induce T-cell dysfunction (so called “T-cell exhaustion”) or apoptosis. Employing this inhibitory pathway, the immune system can attenuate excessive immune reactions and ensure self-tolerance, which is important for maintaining immune homeostasis. These functions involve programmed cell death protein-1 (PD-1), programmed cell death-1 ligand-1/2 (PD-L1/2), cytotoxic T lymphocyte antigen-4 (CTLA-4), lymphocyte-activation gene 3 (LAG-3), T-cell immunoglobulin mucin-3 (TIM-3), and B and T lymphocyte attenuator (BTLA). Tumor cells harness these suppressive effects as one of their “immunoediting” mechanisms [2]. As shown in recent clinical trials, immune checkpoint blockade with monoclonal antibody promotes endogenous antitumor activities of immune cells and achieves clinically significant benefits for cancer patients [3, 4].

In this review, we focus on the current development status of and future challenges in utilizing immune checkpoint inhibitors, especially CTLA-4, PD-1, and PD-L1.

## 2. Anti-CTLA-4 Antibody

CTLA-4 (also known as CD152) is a member of the CD28 family of receptors [21, 22]. CTLA-4 is inducibly expressed on the surfaces of activated conventional CD4^+^ and CD8^+^ T cells. CTLA-4 binds to ligands B7.1 (CD80) and B7.2 (CD86) on APCs, where it competes with costimulatory receptor CD28 to bind with shared ligands. As CTLA-4 binds with higher affinity than CD28, it reduces CD28-dependent costimulation. CTLA-4 also mediates direct inhibitory effects on the MHC-TCR pathway [23]. CTLA-4 recruits 2 phosphatases, SHP-2 and PP2A, to its intracellular YVKM domain. SHP-2 dephosphorylates the CD3*ζ* chain, attenuating the TCR signal. PP2A inhibits downstream Akt phosphorylation, further impairing TCR signaling. Furthermore, CTLA-4 is constitutively and highly expressed on CD4^+^CD25^+^FOXP3^+^ regulatory T cells (T regs) and plays a role in their suppressive functions [24–26]. CTLA-4 knockout mice have a lethal autoimmune-like syndrome. Prominent infiltration of CD4^+^ T cells is detected in multiple organs. Thus, CTLA-4 is considered to be indispensable for maintaining immune homeostasis.

In the tumor microenvironment, CTLA-4 suppresses antitumor immune activities. In animal models, it has been shown that CTLA-4 blockade leads to reactivation of the antitumor immune response and tumor shrinkage [27–29]. The mechanism of action has not yet been fully elucidated. Observations made to date suggest that anti-CTLA-4 antibodies function not only by blocking inhibitory signals from reaching effector T cells but also by depleting regulatory T cells in the tumor microenvironment [30, 31]. For use in humans, based on preclinical studies, two anti-CTLA-4 antibodies have been developed: ipilimumab (Bristol-Myers Squibb) and tremelimumab (Pfizer).

### 2.1. Ipilimumab

Ipilimumab is a fully humanized IgG1 monoclonal antibody that inhibits CTLA-4 [32, 33].

Early clinical trials evaluated ipilimumab in patients with a variety of malignancies, including melanoma, prostate cancer, renal cell carcinoma, and non-Hodgkin lymphoma [34–45]. Some of these studies combined ipilimumab with a peptide vaccine, chemotherapy, or IL-2. Based on preclinical data, ipilimumab was administered at a dose range of 0.1–20 mg/kg, employing single or multiple dosing schedules (every 3-4 weeks).

A phase I study evaluated a single 3 mg/kg dose of ipilimumab for patients with metastatic hormone-refractory prostate cancer. Two (14%) of 14 patients showed ≧50% decline in prostate specific antigen. One (7%) patient developed grade 3 rash/pruritus requiring systemic corticosteroid administration [36]. Another phase I trial combined ipilimumab (administered at 3 mg/kg every 3 weeks) with a glycoprotein (gp) 100 peptide vaccine for patients with metastatic melanoma. Three (21%) of 14 patients responded to this treatment, including 2 showing complete responses (CRs). Grade 3 to 4 immune-related adverse events (irAEs) occurred in 6 (43%) patients. These irAEs included dermatitis, enterocolitis, hepatitis, and hypophysitis [34]. On the whole, irAEs were mild and manageable with therapy discontinuation and/or appropriate treatments, including corticosteroids.

A phase II trial compared 3 doses (0.3, 3, or 10 mg/kg) administered every 3 weeks for a total of 4 doses. Eligible patients were permitted to receive reinduction therapy (at a dose of 10 mg/kg) or maintenance therapy (administered at the previously assigned dose level every 12 weeks). The overall response rate (ORR) in the 10 mg/kg arm was superior to those in the other arms (11.1% versus 4.2% versus 0.0%), but irAEs were also higher in the 10 mg/kg arm [43]. The optimal dosing and scheduling are as yet unknown. A phase III randomized trial (NCT01515189) is currently comparing 2 doses (3 mg/kg versus 10 mg/kg). No consensus has yet been reached on the relative significance of reinduction versus maintenance therapy [46, 47]. A prospective study comparing reinduction therapy versus the physician's choice of chemotherapy (NCT00495066) is currently underway.

Based on pivotal phase III randomized controlled trials (RCTs) showing survival benefit, ipilimumab was approved by the US Food and Drug Administration (FDA) for metastatic melanoma [5, 6]. In the landmark phase III trial for patients with previously treated metastatic melanoma, ipilimumab (administered at 3 mg/kg every 3 weeks for a total of 4 doses) with or without the gp 100 peptide vaccination was compared with the gp 100 peptide vaccine alone. Eligible patients were permitted to receive reinduction therapy. The median OSs in the ipilimumab-containing arms were significantly superior to that in the gp 100 alone arm (10.1 months in ipilimumab/gp 100, 10.0 months in ipilimumab alone, and 6.4 months in gp 100 alone, hazard ratio (HR) 0.68; *P* < 0.001). Grade 3 to 4 irAEs were seen in 10–15% of patients in the ipilimumab-containing arms, while 3% in the gp 100 alone arm experienced irAEs. There were 14 treatment-related deaths (2.1%), including 7 patients with irAEs [5]. Long-term follow-up analysis confirmed an approximately 20% survival rate for patients in the ipilimumab-containing arms. Safety profiles in long-term survivors were comparable among the 3 groups, and new onset irAEs after the last dose of ipilimumab were infrequent (8%; all grades) [48]. The other phase III trial compared ipilimumab (at 10 mg/kg every 3 weeks for 4 doses)/dacarbazine with dacarbazine/placebo, followed by maintenance therapy with ipilimumab or placebo administered every 12 weeks for eligible patients. Overall survival (OS) was significantly longer in the ipilimumab/dacarbazine arm (11.2 versus 9.1 months), and the higher survival rates were durable (47.3% versus 36.3% at 1 year, 28.5% versus 17.9% at 2 years, 20.8% versus 12.2% at 3 years, HR for death 0.72; *P* < 0.001). Grade 3 to 4 AEs were seen in more patients in the ipilimumab/dacarbazine arm (56.3% versus 27.5%; *P* < 0.001). No drug-related deaths occurred among those in the ipilimumab/dacarbazine arm [6].

The analysis of the collected data from 12 previous clinical trials, which include 1861 ipilimumab-treated patients with advanced melanoma, demonstrated a median OS of 11.4 months and 3-year OS rate of 22%. The OS curve started to show plateau around year 3, which was independent of the dose of ipilimumab (3 or 10 mg/kg), therapy line (treatment-naïve or not), or use of maintenance therapy [49].

### 2.2. Tremelimumab

Tremelimumab is a human IgG2 monoclonal antibody that blocks CTLA-4 [50].

Early clinical trials on tremelimumab monotherapy showed response rates of 2–17%, and these responses were durable (>150 days) [51–57]. Based on preclinical and clinical data, the standard regimen is 15 mg/kg every 90 days. Most adverse events were mild and manageable. These adverse events included skin rash, diarrhea, and endocrine abnormalities.

A phase III study compared tremelimumab (15 mg/kg every 3 months) with chemotherapy (physician's choice) in patients with untreated advanced melanoma [7]. This study demonstrated no benefits in either ORR (10.7% versus 9.8%) or OS (12.6 mo versus 10.7 mo), but a superior response duration was seen (35.8 versus 13.7 months). This observation might be explained by patient selection bias (exclusion of patients with lactate dehydrogenase (LDH) >2x upper limit of normal), drug crossover (to ipilimumab) in the control arm, and even a potentially suboptimal dosing regimen. Tremelimumab is still being investigated for other tumors, both alone and as combination therapy (Table 1).

## 3. Anti-PD-1 Antibodies

Programmed cell death protein-1 (PD-1; also known as CD279), like CTLA-4, is a coinhibitory CD28-family molecule [22]. While CTLA-4 works in the early phase of naïve-T-cell activation, PD-1 functions mainly in the late phase, in which PD-1 induces exhaustion or anergy in effector T cells. Thus, PD-1 is considered to play an important role in chronic inflammation such as that associated with viral infection or tumor exposure [58]. PD-1 is expressed on activated T cells, T regs [59], activated B cells, NK cells, and monocytes. It binds to the B7-family ligands PD-L1 (programmed death ligand-1, B7-H1) and PD-L2 (programmed death ligand-2, B7-DC) on APCs. PD-1 has cytoplasmic domain motifs known as ITIM (immunoreceptor tyrosine-based inhibitory motif) and ITSM (immunoreceptor tyrosine-based switch motif) [23]. When these motifs are phosphorylated, they recruit two inhibitory phosphatases, SHP-1 and SHP-2 (SHP: SH2-containing-phosphatase). These phosphatases dephosphorylate the CD3*ζ* chain, decreasing TCR signaling. Although the inhibitory mechanisms of CTLA-4 and PD-1 have some similarity in terms of inhibiting Akt activation, CTLA-4 can also interfere with Akt independently via PP2A [23]. PD-1 knockout mice show a milder lupus-like syndrome than CTLA-4 knockout mice [60].

Tumor cells utilize the PD-1-PD-L1/2 pathway to evade immune-cell attack [61]. Blockade of this pathway was shown to restore and augment antitumor immune activities [62].

### 3.1. Nivolumab (BMS-936558/ONO-4538)

Nivolumab is a fully humanized IgG4 monoclonal antibody that blocks PD-1 [62].

Phase I studies tested nivolumab in such cancers as melanoma, non-small cell carcinoma of the lung (NSCLC), ovarian cancer, and renal cell carcinoma. These studies showed response rates of approximately 20–30%, durable tumor regression (>1 year), and an acceptable safety profile, with Grade 3 to 4 irAEs developing in about 20% of patients [8, 9, 63–65]. In long-term follow-up of the phase I trial for advanced melanoma, median OS was 16.8 months and survival rates were 62% at 1 year and 43% at 2 years. The patients requiring discontinuation of treatment maintained their tumor responses for at least 16 months (16–56 months). Long-term safety profiles were acceptable and similar to those described in a previous report [8]. The preliminary results of a phase I study evaluating nivolumab (at 3 mg/kg q2w) for untreated advanced NSCLC were recently reported. The ORR was 30% with 2 complete remissions (CRs), as measured by RECIST. ORR and progression-free survival (PFS) correlated with PD-L1 positivity (67% versus 0% for ORR, 45.6 mo versus 36.1 mo for median PFS). AEs were generally manageable and grade 3 to 4 AEs occurred in 3 patients, including rash, increased transaminase, and hyperglycemia [66].

Recently the interim analysis report of a phase III study (NCT01721746), comparing nivolumab monotherapy (at 3 mg/kg q2w) with investigator's choice chemotherapy in ipilimumab-refractory advanced melanoma, was shown. The ORRs were 32% in the nivolumab arm and 11% in the control arm, with the median duration of response in the nivolumab arm not reached. Grade 3 to 4 drug-related AEs were less frequent in the nivolumab arm (9% versus 31%) [10]. Another phase III study (NCT01721772) compared nivolumab monotherapy (at 3 mg/kg q2w) with dacarbazine in 418 patients with previously untreated stage III or IV melanoma. This study was stopped ahead of schedule and unblinded after independent data monitoring committee found significant survival superiority in nivolumab over dacarbazine. The results from the double-blind part of the study before the stoppage showed that the OS rate at 1 year was significantly higher in the nivolumab arm (72.9% versus 42.1%, HR for death 0.42; *P* < 0.001), and the median PFS was also significantly longer in the nivolumab arm (5.1 versus 2.2 months, HR for death or progression 0.43; *P* < 0.001). Grade 3 to 4 drug-related AEs occurred in more patients in the dacarbazine arm (11.7% versus 17.6%). No drug-related deaths occurred in both arms [11]. A phase II study (NCT01927419) of nivolumab in combination with ipilimumab compared with ipilimumab alone for advanced melanoma is currently ongoing (recruitment has been completed).

In 2013, nivolumab received Fast Track designation for the treatment of NSCLC, melanoma, and renal cell carcinoma (RCC) from the FDA. In April 2014, a rolling submission to the FDA for nivolumab in third-line pretreated NSCLC was started. In May 2014, nivolumab received a Breakthrough Therapy designation for non-Hodgkin lymphoma from the FDA. In Japan, in July 2014, nivolumab received manufacturing and marketing approval for unresectable melanoma from the domestic regulator, the Ministry of Health Labor and Welfare, which made nivolumab the first in anti-PD-1 antibody to receive regulatory approval in the world.

### 3.2. Pidilizumab (CT-011)

Pidilizumab (CT-011) is a humanized IgG-1*κ* monoclonal antibody that blocks PD-1. In animal models, an antitumor effect was achieved with BAT monoclonal antibody (a murine mAb developed against a membrane preparation of a Burkitt lymphoma cell line), from which pidilizumab is derived [67, 68].

In humans, the safety and tolerability of the single dose regimen were shown in a phase I study of patients with advanced hematologic malignancies [69]. No treatment-related toxicities occurred and the maximum tolerated dose was not identified in this trial (0.2–6 mg/kg).

Pidilizumab has been tested in phase II trials, as monotherapy for patients with diffuse large B-cell lymphoma after autologous hematopoietic stem-cell transplantation [70] and as combined therapy with rituximab for relapsed follicular lymphoma [71]. Both trials showed promising efficacies even in high-risk patients.

The results of a phase II trial in patients with pretreated advanced melanoma were recently reported. ORR was 5.9%, measured by immune-related response criteria (irRC), and the OS rate at 1 year was 64.5%. The patients who had been pretreated with ipilimumab (51% of patients) tended to experience a higher rate of immune-related stable disease (irSD) and longer PFS (2.8 mo versus 1.9 mo) [12].

### 3.3. Pembrolizumab (MK-3475, Formally Known as Lambrolizumab)

Pembrolizumab (MK-3475) is a humanized monoclonal IgG-4*κ* antibody that blocks PD-1.

A phase I dose-escalation study evaluated three dose levels, 1 mg/kg, 3 mg/kg, and 10 mg/kg, administered every 2 weeks, in patients with multiple solid tumors [72]. All dose levels were found to be safe, and the maximum tolerated dose was not identified. Clinical responses were observed at all dose levels. Another phase I study tested 3 regimens (2 mg/kg every 3 weeks and 10 mg/kg every 2 or 3 weeks) in patients with advanced melanoma [13]. AEs were generally mild and grade 3 to 4 AEs were seen in 13% of patients. The ORRs ranged from 38% to 52%, in the biweekly 10 mg/kg cohort (measured by RECIST), showing no significant differences. These responses were durable, with the median PFS exceeding 7 months for all three regimens.

An ongoing phase II trial is now comparing 2 dose levels of pembrolizumab with investigator-choice chemotherapy in patients with previously treated advanced melanoma (NCT01704287). Another ongoing phase II trial is also evaluating 2 dose schedules of pembrolizumab (10 mg/kg q2w or q3w) compared with ipilimumab (3 mg/kg q3w) for advanced melanoma (NCT01866319).

In April 2013, pembrolizumab received the Breakthrough Therapy designation for advanced melanoma from the FDA. After being reviewed under the FDA's Accelerated Approval program, in September 2014, pembrolizumab received approval for treatment of patients with advanced melanoma by the FDA.

Besides melanoma, several early trials have showed the tolerability and antitumor effects of pembrolizumab in other tumors. The preliminary results of another phase I study evaluating pembrolizumab in untreated PD-L1-positive NSCLC were recently reported. The overall objective response rate was 25% (33% in the 2 mg/kg q3w, 20% in the 10 mg/kg q3w, and 31% in the 10 mg/kg q2w group), as measured by RECIST. AEs were generally mild and grade 3 to 4 AEs occurred in 3 patients, including pneumonitis requiring treatment discontinuation [14]. Another preliminary result was reported for the phase I trial of pembrolizumab as monotherapy, administered at 2 mg/kg every 2 weeks, to 60 patients with recurrent/metastatic head and neck cancers. Grade 3 to 4 drug-related AEs were reported in 16.7% of patients. The best ORR was 20% in all patients (assessed by RECIST 1.1). Efficacies were comparable between human papilloma virus- (HPV-) positive and HPV-negative patients (20.0% versus 19.4%) [15]. Another phase I study (NCT01848834) assessed pembrolizumab in the patients with previously treated advanced gastric cancer that expressed PD-L1. The enrolled 39 patients were treated with pembrolizumab at 10 mg/kg q2w. Median follow-up period was 6 months. Treatment-related AEs occurred in 24 patients (61.5%), and those of grade 3 to 5 occurred in 3 patients (pneumonitis, peripheral neuropathy, and hypoxia). ORR was 30.8% and disease control rate was 43.6%. Responses were mostly ongoing and the median response duration was not reached [16].

## 4. Anti-PD-L1 Antibodies

PD-L1 (also known as B7-H1 or CD274) and PD-L2 (also known as B7-DC or CD273) are inhibitory B7-family molecules that bind the PD-1 receptor. PD-L1 is inducibly expressed on a variety of hematopoietic and nonhematopoietic cells, including most human tumor cells and cells within the tumor microenvironment [61]. PD-L1 expression has been shown to correlate inversely with the clinical outcomes of some malignancies. PD-L2 is expressed on hematopoietic cells. PD-L1 knockout mice show infiltration of lymphocytes into nonlymphoid organs and exacerbation of preexisting autoimmune diseases [73, 74].

As mentioned above, the PD-1-PD-L1 axis is one of the main mechanisms by which cancer cells evade immune-cell attack [61]. Blockade of this pathway was shown to reinforce antitumor immune activities [62]. Because PD-L1 also interacts with CD80 [75, 76], anti-PD-L1 antibody might have optimal clinical potency against PD-1.

### 4.1. BMS-936559

BMS-936559 is a fully humanized IgG4 monoclonal anti-PD-L1 antibody. It inhibits the binding of PD-L1 to PD-1 and CD80. A phase I dose-escalation study evaluated BMS-936559 in 207 patients with selected cancers, including melanoma, NSCLC, ovarian cancer, and renal cell carcinoma. The study drug was administered at 4 dose levels (0.3–10 mg/kg) every 14 days, 3 times in each 6-week course for up to 16 cycles, when either CR or disease progression was confirmed. The ORRs were 6–17% and efficacy was durable (>1 year in 8 of 16 patients who responded). Grade 3 to 4 irAEs, seen in 9% of the patients, were treatment-related in 5% [17].

### 4.2. MPDL3280A

MPDL3280A is a humanized IgG-1*κ* monoclonal anti-PD-L1 antibody. It is genetically engineered to modify the Fc domain, thereby impairing the antibody-dependent cellular cytotoxicity of PD-L1 expressing cells [77, 78].

A phase I trial of MPDL3280A as monotherapy for advanced melanoma achieved a response rate of 26% and PFS of 35% at 24 weeks. Grade 3 to 4 AEs were seen in 33% of patients [79]. The results of another phase I trial were recently reported. MPDL3280A was tested in patients with pretreated metastatic urothelial bladder cancer. ORR in PD-L1-positive patients was superior to that in PD-L1-negative patients (43% versus 11% at 6 weeks). ORR at 12 weeks was 52% in PD-L1-positive patients. Grade 3 to 4 AEs were seen in 4% of patients, with no irAEs [18]. The FDA has granted the Breakthrough Therapy designation to MPDL3280A.

### 4.3. MEDI4736

MEDI4736 is a humanized IgG-1*κ* monoclonal antibody that blocks PD-L1. MEDI4736 demonstrated tumor regression and improved survival in a mouse model.

A “first-time-in-human” phase I study evaluating the safety, tolerability, and pharmacokinetics of this agent in patients with advanced solid tumors is currently underway (NCT01693562). The interim report was recently presented. As of January 2014, 26 patients were receiving dose-escalation treatments and had been given a median of 5 (1–25) q2w and 4.5 (1–7) q3w doses of MEDI4736 across 6 cohorts (0.1–10 mg/kg q2w; 15 mg/kg q3w). No dose limiting toxicities (DLTs) or maximum tolerated dose was identified. Treatment-related AEs occurred in 34% of patients, but all were grade 1 to 2 and did not lead totreatment discontinuation. Four of the 26 patients showed partial responses (PRs). The rate (PR + stable disease ≧ 12 weeks) was 46%. Clinical responses were durable, with 11 patients remaining in the study (2+ to 14.9+ months) [19]. Another phase I trial is now testing the combination of MEDI4736 plus tremelimumab (NCT01975831).

### 4.4. MSB0010718C

MSB0010718 is a fully humanized IgG1 monoclonal antibody directed to PD-L1. A phase I trial is currently testing MSB0010718 to assess its safety, tolerability, and pharmacokinetics in patients with refractory malignancies (NCT01772004). As of January 2014, 27 patients had been enrolled and were participating in a dose-escalation study (3 + 3 design; 1, 3, 10, and 20 mg/kg, q2w). Twenty-three patients had been followed for at least 4 weeks. Discontinuation of the treatment had been necessary in 12 patients (52.2%): 9 (39.1%) due to progression of disease, 2 (8.7%) for AEs, and 1 (4.3%) because the patient died. Grade 3 to 4 drug-related toxicities included laboratory abnormalities in 3 patients. One DLT was observed in 1 patient at dose level 4 (20 mg/kg): an irAE with creatine kinase elevation, myositis, and myocarditis [20].

## 5. Combination Therapy

Recent clinical trials have actively investigated the potential for synergistic effects by combining immune checkpoint inhibitors with other agents. The partner agents/therapies include other checkpoint agents, cytotoxic agents, anticancer vaccines, cytokines, and radiotherapy.

A phase I study evaluated combined therapy with ipilimumab plus nivolumab in patients with advanced melanoma [80]. The patients received ipilimumab once every 3 weeks for 4 doses and nivolumab once every 3 weeks for 8 doses concurrently. Then, eligible patients were permitted to receive both once every 12 weeks up to 8 doses. Grade 3 to 4 treatment-related AEs were seen in 53% of the concurrent-cohort patients but were mild and manageable. The maximum tolerated dose was 3 mg/kg of ipilimumab and 1 mg/kg of nivolumab, a dosing regimen at which 53% of patients showed responses. Recent follow-up surveys confirmed OS to be 94% at 1 year and 88% at 2 years in this cohort. An expansion cohort, with the patients receiving 3 mg/kg of ipilimumab and 1 mg/kg of nivolumab every 3 weeks for 4 doses and 1 mg/kg of nivolumab every 2 weeks until disease progression, is currently being evaluated in a phase II/III study [81]. A phase III trial (NCT01844505) evaluating this combination is currently ongoing (recruitment has been completed).

## 6. Biomarkers for Predicting Clinical Benefits and Adverse Reactions

Although immune checkpoint inhibitors have shown promising safety and efficacy, to date only a small proportion of patients have achieved long-term survival, with severe irAEs occurring on occasion. Biomarkers predicting clinical benefit may enable physicians to select individualized treatments for their patents and thereby maximize clinical benefits. Thus, there is an urgent need to identify “baseline (pretreatment)” biomarkers predicting responses or toxicities. Several biomarkers for examining T-cell proliferation or activation and other forms of antigen-specific immunity have been assessed in the context of immune checkpoint inhibitors.

Immunohistochemical PD-L1 expression in a tumor specimen is among the potential markers for PD-1-PD-L1-directed therapies. In a phase I study of nivolumab, though the data obtained are preliminary, an objective response was seen only in the patients who showed immunohistochemical PD-L1 expression in pretreatment tumor specimens [63]. These observations may support the strategy of selecting PD-L1-positive patients for therapy. However, PD-L1 expression on tumor cells is inducible and is susceptible to influences of the tumor microenvironment. Furthermore, technical advances in PD-L1 immunostaining are still needed. Also, the value of PD-L1 IHC staining as a predictive biomarker for combination therapy with nivolumab plus ipilimumab has yet to be validated [80]. As yet, the applicability and significance of PD-L1 expression as a baseline biomarker must be interpreted with caution and further prospective evaluations are needed, including the results of ongoing randomized clinical trials that are prospectively evaluating PD-L1 IHC as a companion diagnostic platform (NCT01721746).

Another potential biomarker is pretreatment levels of monocytic myeloid-derived suppressor cells (m-MDSCs) [82, 83]. A recent retrospective study suggested higher pretreatment quantities of Lin^−^CD14^+^HLA-DR^low/−^ m-MDSC to be associated with inferior OS in patients with metastatic melanoma treated with ipilimumab [83].

Recent genetic analysis using whole-exome sequencing showed the significance of somatic mutational load as predictive biomarker of clinical benefit in melanoma patients treated with CTLA-4 blockade. The neopeptide signature associated with clinical response was identified and predicted mutant peptides were verified to activate patient T cell* in vitro *[84].

Other potential predictive/prognostic biomarkers include the gene expression profiles obtained employing tumor biopsies [85, 86], CRP level [87], absolute lymphocyte and eosinophil counts [88], and LDH levels [89]. These possibilities await further research.

## 7. Conclusion

Immune checkpoint inhibitors have opened a new era of cancer immunotherapy. Since the FDA approval was obtained for the anti-CTLA-4 monoclonal antibody ipilimumab, several large-scale clinical trials have evaluated new agents both alone and in combinations with other conventional or new therapies. Future challenges include exploring new target molecules and immune cells, optimizing dosing regimens and combination therapies, validating the safety and efficacy of these novel treatment strategies in many other malignancies, establishing an immunomonitoring system to be applied during therapy, and identifying biomarkers predicting clinical responses and toxicities. Active, ongoing investigations are anticipated to provide further clinical benefits for patients with cancers that are currently refractory to treatment.

## Figures and Tables

**Table 1:** 

Target molecule	Drug name	Phase	Status/NCT number	Disease	Number of patients	Study design	Response	Survival	Treatment-related adverse events (≧Gr3)	Reference
CTLA-4	Ipilimumab	III	Completed (NCT00094653)	Melanoma	676	Endpoint: safety/efficacy Ipi + gp100 versus Ipi versus gp100	Ipi + gp100: ORR 5.7%; SD 14.4%	Ipi + gp100 versus gp100: 10.1 versus 6.4 mos	Ipi + gp100: drug-related 17.4%; irAEs 10.2%; diarrhea 4.5%; fatigue 5.0%; dyspnea 3.7%; anemia 2.9%; endocrine abnl. 11%; AST↑ 0.5%; ALT↑ 0.3%	[5]
		III	Completed (NCT00324155)	Melanoma	502	Endpoint: efficacy Ipi + DTIC versus PBO + DITC	Ipi + DTIC: ORR 15.2%; SD 18.0%	Ipi + DTIC versus PBO + DTIC: 11.2 versus 9.1 mos	Ipi + DTIC: immune-related 41.7%; pruritus 2.0%; rash 1.2%; diarrhea 4.0%; colitis 6.1%; AST↑ 17.4%; ALT↑ 20.7%	[6]
	Tremelimumab	III	Completed (NCT00257205)	Melanoma	655	Endpoint: efficacy treme. versus chemo.	ORR 10.7%	Treme. versus chemo.: 12.6 versus 10.7 mos (NS)	52%; diarrhea/colitis 18%; fatigue 6%; rash 2%; pruritus 1%; dyspnea 3%; hypothalamus and pituitary disorders 1%; hepatitis 1%	[7]

PD-1	Nivolumab (BMS-936558/ONO-4538)	I	Ongoing (not recruiting) (NCT00730639)	Melanoma	107	Endpoint: safety/efficacy 5 dosing regimens	ORR 30.8%; median duration of response 104 wks; SD (≧24 wks) 6.5%	OS 16.8 mos; PFS 3.7 mos	22.4%; fatigue 1.9%; diarrhea 1.9%; abdominal pain 1.9%; lymphopenia 2.8%	[8]
		I	Ongoing (not recruiting) (NCT01176461)	Melanoma	90	Endpoint: safety/efficacy 3 dosing regimens	ORR 25%; SD (≧24 wks) 21%	PFS (at 24 wks) 46%	5.6%; rash 2.2%; interstitial pneumonitis 2.2%	[9]
		III	Ongoing (not recruiting) (NCT 01721772)	Melanoma	370	Endpoint: efficacy Nivo. versus ICC	ORR 32% versus 11%	NA	9% versus 31%	[10]
		III	Completed (NCT01721772)	Melanoma	418	Endpoint: efficacy Nivo. versus dacarbazine	ORR 40.0% versus 13.9%	OS (at 1 yr) 72.9% versus 42.1%, median PFS 5.1 versus 2.2 mo	11.7% versus 17.6%; fatigue 0.5%; diarrhea 1.0%; rash 0.5%; vomiting 0.5%	[11]
	Pidilizumab (CT-011)	II	Completed (NCT01435369)	Melanoma	103	Endpoint: safety/efficacy 2 dosing regimens	ORR 5.9%	OS (at 1 yr): 64.5%	NA	[12]
	Pembrolizumab (MK-3475)	I	Ongoing (not recruiting) (NCT01295827)	Melanoma	135	Endpoint: safety/efficacy 3 dosing regimens	ORR 38% by RECIST and 37% by irRC	Median PFS >7 mos	13%; hypothyroidism 1%; diarrhea 1%; fatigue 1%; AST↑ 1%; renal failure 1%; rash 2%; pruritus 1%	[13]
		I	Ongoing (not recruiting) (NCT01295827)	Untreated NSCLC	57	Endpoint: safety/efficacy 3 dosing regimens	ORR 26% by RECIST and 47% by irRC	Median OS NR; OS at 1 yr 80%; median PFS 45.6%; PFS at 24 wks 70%	CK↑ 2%; pericardial effusion 2%; pneumonitis 2%; acute kidney injury 2%	[14]
		I	Ongoing (not recruiting) (NCT01848834)	Head and neck cancer	60	Endpoint: safety/efficacy single arm	ORR 19.6% in total, 20.0% in HPV+, and 19.4% in HPV−;	NA	Gr3–5 16.7%; Rash 3.3%	[15]
		I	Ongoing (not recruiting) (NCT01848834)	Gastric cancer	39	Endpoint: safety/efficacy single arm	ORR 30.2% by RECIST	NA	7.7%; hypoxia 2.6%; peripheral neuropathy 2.6%; pneumonia 2.6%	[16]

PD-L1	BMS-936559	I	Ongoing (not recruiting) (NCT00729664)	Melanoma	52	Endpoint: safety 4 dose levels	ORR 17%; SD (≧24 wks) 27%	PFS (at 24 wks) 42%	9%; fatigue 1%; infusion reaction 1%; lymphopenia 1%	[17]
				NSCLC	49		ORR 10%; SD (≧24 wks) 12%	PFS (at 24 wks) 31%		
				Ovarian cancer	17		ORR 6%; SD (≧24 wks) 18%	PFS (at 24 wks) 22%		
				Renal cell carcinoma	17		ORR 12%; SD (≧24 wks) 41%	PFS (at 24 wks) 53%		
	MPDL3280A	I	Recruiting (NCT01375842)	Urothelial bladder cancer	68	Endpoint: safety/efficacy/ biomarker single arm	ORR: PD-L1 + 43% (at 6 wks) and 52% (at 12 wks); PD-L1 − 11% (at 6 wks);	NA	4%; no irAE	[18]
	MEDI4736	I	Recruiting (NCT01693562)	Advanced solid tumors	26 (as of Jan 2014)	Endpoint: safety/efficacy single arm	PR 15.4%; disease control rate (≧12 wks) 46%	NA	Any Gr 34%; Gr3/4 0%; no DLT; no MTD	[19]
	MSB0019718C	I	Recruiting (NCT01772004)	Refractory malignancies	27 (as of Jan 2014)	Endpoint: safety single arm	NA	NA	Treatment discontinuation 52.2% (8.7% for AEs); drug-related AEs 11.1%; DLT 3.7% (CPK↑, myositis, and myocarditis)	[20]

Abbreviations: NSCLC, non-small cell lung cancer; Ipi, Ipilimumab; gp100, glycoprotein 100 peptide vaccine; DITC, dacarbazine; PBO, placebo; ORR, objective response rate; PR, partial response; SD, stable disease; mo, month; wk, week; RECIST, response evaluation criteria in solid tumors; irRC, immune-related response criteria; HPV, human papillomavirus; NA, not available; NS, not significant; NR, not reached; OS, overall survival; PFS, progression-free survival; AE, adverse event; irAE, immune-related adverse event; Gr, Grade; abnl., abnormality; AST, aspartate aminotransferase; ALT, alanine aminotransferase; CPK, creatine phosphokinase; DLT, dose limiting toxicity; MTD, maximum tolerance dose; ICC, investigator's choise chemotherapy.

## References

[1] Chen L., Flies D. B. (2013). Molecular mechanisms of T cell co-stimulation and co-inhibition. *Nature Reviews Immunology*.

[2] Schreiber R. D., Old L. J., Smyth M. J. (2011). Cancer immunoediting: integrating immunity's roles in cancer suppression and promotion. *Science*.

[3] Page D. B., Postow M. A., Callahan M. K., Allison J. P., Wolchok J. D. (2014). Immune modulation in cancer with antibodies. *Annual Review of Medicine*.

[4] Pardoll D. M. (2012). The blockade of immune checkpoints in cancer immunotherapy. *Nature Reviews Cancer*.

[5] Hodi F. S., O'Day S. J., McDermott D. F. (2010). Improved survival with ipilimumab in patients with metastatic melanoma. *The New England Journal of Medicine*.

[6] Robert C., Thomas L., Bondarenko I. (2011). Ipilimumab plus dacarbazine for previously untreated metastatic melanoma. *The New England Journal of Medicine*.

[7] Ribas A., Kefford R., Marshall M. A. (2013). Phase III randomized clinical trial comparing tremelimumab with standard-of-care chemotherapy in patients with advanced melanoma. *Journal of Clinical Oncology*.

[8] Topalian S. L., Sznol M., McDermott D. F. (2014). Survival, durable tumor remission, and long-term safety in patients with advanced melanoma receiving nivolumab. *Journal of Clinical Oncology*.

[9] Weber J. S., Kudchadkar R. R., Yu B. (2013). Safety, efficacy, and biomarkers of nivolumab with vaccine in ipilimumab-refractory or -naive melanoma. *Journal of Clinical Oncology*.

[10] Weber J. S., D'Angelo S., Hodi F. S. A phase 3 randomized, open-label study of nivolumab (anti-PD-1; BMS-936558; ONO-4538) versus investigator's choice chemotherapy (ICC) in patients with advanced melanoma after prior anti-CTLA-4 therapy.

[11] Robert C., Long G. V., Brady B. (2014). Nivolumab in previously untreated melanoma without BRAF mutation. *The New England Journal of Medicine*.

[12] Atkins M. B., Kudchadkar R. R., Sznol M. Phase 2, multicenter, safety and efficacy study of pidilizumab in patients with metastatic melanoma.

[13] Hamid O., Robert C., Daud A. (2013). Safety and tumor responses with lambrolizumab (anti-PD-1) in melanoma. *The New England Journal of Medicine*.

[14] Rizvi N. A., Garon E. B., Patnaik A. (2014). Safety and clinical activity of MK-3475 as initial therapy in patients with advanced non-small cell lung cancer (NSCLC). *Proceedings of the ASCO Meeting Abstracts*.

[15] Seiwert T. Y., Burtness B., Weiss J. (2014). A phase Ib study of MK-3475 in patients with human papillomavirus (HPV)-associated and non-HPV-associated head and neck (H/N) cancer. *ASCO Meeting Abstracts*.

[16] Muro K., Bang Y., Shankaran V. A phase 1b study of pembrolizumab (Pembro; MK-3475) in patients (Pts) with advanced gastric cancer.

[17] Brahmer J. R., Tykodi S. S., Chow L. Q. M. (2012). Safety and activity of anti-PD-L1 antibody in patients with advanced cancer. *The New England Journal of Medicine*.

[18] Powles T., Vogelzang N. J., Fine G. D. (2014). Inhibition of PD-L1 by MPDL3280A and clinical activity in pts with metastatic urothelial bladder cancer (UBC). *ASCO Meeting Abstracts*.

[19] Lutzky J., Antonia S. J., Blake-Haskins A. (2014). A phase 1 study of MEDI4736, an anti–PD-L1 antibody, in patients with advanced solid tumors. *ASCO Meeting Abstracts*.

[20] Heery C. R., O'Sullivan Coyne G. H., Madan R. A. Phase I open-label, multiple ascending dose trial of MSB0010718C, an anti-PD-L1 monoclonal antibody, in advanced solid malignancies.

[21] Rudd C. E., Taylor A., Schneider H. (2009). CD28 and CTLA-4 coreceptor expression and signal transduction. *Immunological Reviews*.

[22] Chen L. (2004). Co-inhibitory molecules of the B7-CD28 family in the control of T-cell immunity. *Nature Reviews Immunology*.

[23] Nirschl C. J., Drake C. G. (2013). Molecular pathways: coexpression of immune checkpoint molecules: Signaling pathways and implications for cancer immunotherapy. *Clinical Cancer Research*.

[24] Sansom D. M., Walker L. S. K. (2006). The role of CD28 and cytotoxic T-lymphocyte antigen-4 (CTLA-4) in regulatory T-cell biology. *Immunological Reviews*.

[25] Wing K., Onishi Y., Prieto-Martin P. (2008). CTLA-4 control over Foxp3^+^ regulatory T cell function. *Science*.

[26] Walker L. S. K. (2013). Treg and CTLA-4: two intertwining pathways to immune tolerance. *Journal of Autoimmunity*.

[27] Leach D. R., Krummel M. F., Allison J. P. (1996). Enhancement of antitumor immunity by CTLA-4 blockade. *Science*.

[28] van Elsas A., Hurwitz A. A., Allison J. P. (1999). Combination immunotherapy of B16 melanoma using anti-cytotoxic T lymphocyte-associated antigen 4 (CTLA-4) and granulocyte/macrophage colony- stimulating factor (GM-CSF)-producing vaccines induces rejection of subcutaneous and metastatic tumors accompanied by autoimmune depigmentation. *Journal of Experimental Medicine*.

[29] Quezada S. A., Simpson T. R., Peggs K. S. (2010). Tumor-reactive CD4^+^ T cells develop cytotoxic activity and eradicate large established melanoma after transfer into lymphopenic hosts. *The Journal of Experimental Medicine*.

[30] Peggs K. S., Quezada S. A., Chambers C. A., Korman A. J., Allison J. P. (2009). Blockade of CTLA-4 on both effector and regulatory T cell compartments contributes to the antitumor activity of anti-CTLA-4 antibodies. *The Journal of Experimental Medicine*.

[31] Simpson T. R., Li F., Montalvo-Ortiz W. (2013). Fc-dependent depletion of tumor-infiltrating regulatory t cells co-defines the efficacy of anti-CTLA-4 therapy against melanoma. *The Journal of Experimental Medicine*.

[32] Hoos A., Ibrahim R., Korman A. (2010). Development of ipilimumab: contribution to a new paradigm for cancer immunotherapy. *Seminars in Oncology*.

[33] Lipson E. J., Drake C. G. (2011). Ipilimumab: an anti-CTLA-4 antibody for metastatic melanoma. *Clinical Cancer Research*.

[34] Phan G. Q., Yang J. C., Sherry R. M. (2003). Cancer regression and autoimmunity induced by cytotoxic T lymphocyte-associated antigen 4 blockade in patients with metastatic melanoma. *Proceedings of the National Academy of Sciences of the United States of America*.

[35] Maker A. V., Phan G. Q., Attia P. (2005). Tumor regression and autoimmunity in patients treated with cytotoxic T lymphocyte-associated antigen 4 blockade and interleukin 2: a phase I/II study. *Annals of Surgical Oncology*.

[36] Small E. J., Tchekmedyian N. S., Rini B. I., Fong L., Lowy I., Allison J. P. (2007). A pilot trial of CTLA-4 blockade with human anti-CTLA-4 in patients with hormone-refractory prostate cancer. *Clinical Cancer Research*.

[37] Ansell S. M., Hurvitz S. A., Koenig P. A. (2009). Phase I study of ipilimumab, an anti-CTLA-4 monoclonal antibody, in patients with relapsed and refractory B-cell non-Hodgkin lymphoma. *Clinical Cancer Research*.

[38] Fong L., Kwek S. S., O'Brien S. (2009). Potentiating endogenous antitumor immunity to prostate cancer through combination immunotherapy with CTLA4 blockade and GM-CSF. *Cancer Research*.

[39] Hodi F. S., Butler M., Oble D. A. (2008). Immunologic and clinical effects of antibody blockade of cytotoxic T lymphocyte-associated antigen 4 in previously vaccinated cancer patients. *Proceedings of the National Academy of Sciences of the United States of America*.

[40] Yang J. C., Hughes M., Kammula U. (2007). Ipilimumab (anti-CTLA4 antibody) causes regression of metastatic renal cell cancer associated with enteritis and hypophysitis. *Journal of Immunotherapy*.

[41] Weber J., Thompson J. A., Hamid O. (2009). A randomized, double-blind, placebo-controlled, phase II study comparing the tolerability and efficacy of ipilimumab administered with or without prophylactic budesonide in patients with unresectable stage III or IV melanoma. *Clinical Cancer Research*.

[42] O'Day S. J., Maio M., Chiarion-Sileni V. (2010). Efficacy and safety of ipilimumab monotherapy in patients with pretreated advanced melanoma: a multicenter single-arm phase II study. *Annals of Oncology*.

[43] Wolchok J. D., Neyns B., Linette G. (2010). Ipilimumab monotherapy in patients with pretreated advanced melanoma: a randomised, double-blind, multicentre, phase 2, dose-ranging study. *The Lancet Oncology*.

[44] Hersh E. M., O'Day S. J., Powderly J. (2011). A phase II multicenter study of ipilimumab with or without dacarbazine in chemotherapy-naïve patients with advanced melanoma. *Investigational New Drugs*.

[45] Margolin K., Ernstoff M. S., Hamid O. (2012). Ipilimumab in patients with melanoma and brain metastases: an open-label, phase 2 trial. *The Lancet Oncology*.

[46] Robert C., Schadendorf D., Messina M., Hodi F. S., O'Day S. (2013). Efficacy and safety of retreatment with ipilimumab in patients with pretreated advanced melanoma who progressed after initially achieving disease control. *Clinical Cancer Research*.

[47] Chiarion-Sileni V., Pigozzo J., Ascierto P. A. (2014). Ipilimumab retreatment in patients with pretreated advanced melanoma: the expanded access programme in Italy. *British Journal of Cancer*.

[48] McDermott D., Haanen J., Chen T.-T., Lorigan P., O'Day S. (2013). Efficacy and safety of ipilimumab in metastatic melanoma patients surviving more than 2 years following treatment in a phase III trial (MDX010-20). *Annals of Oncology*.

[49] Schadendorf D., Robert C., Weber J. S. (2013). Pooled analysis of long-term survival data from phase II and phase III trials of ipilimumab in metastatic or locally advanced, unresectable melanoma. *European Journal of Cancer*.

[50] Ribas A., Hanson D. C., Noe D. A. (2007). Tremelimumab (CP-675,206), a cytotoxic T lymphocyte—associated antigen 4 blocking monoclonal antibody in clinical development for patients with cancer. *The Oncologist*.

[51] Ribas A., Camacho L. H., Lopez-Berestein G. (2005). Antitumor activity in melanoma and anti-self responses in a phase I trial with the anti-cytotoxic T lymphocyte-associated antigen 4 monoclonal antibody CP-675,206. *Journal of Clinical Oncology*.

[52] Camacho L. H., Antonia S., Sosman J. (2009). Phase I/II trial of tremelimumab in patients with metastatic melanoma. *Journal of Clinical Oncology*.

[53] Ribas A., Comin-Anduix B., Chmielowski B. (2009). Dendritic cell vaccination combined with CTLA4 blockade in patients with metastatic melanoma. *Clinical Cancer Research*.

[54] Chung K. Y., Gore I., Fong L. (2010). Phase II study of the anti-cytotoxic T-lymphocyte-associated antigen 4 monoclonal antibody, tremelimumab, in patients with refractory metastatic colorectal cancer. *Journal of Clinical Oncology*.

[55] Kirkwood J. M., Lorigan P., Hersey P. (2010). Phase II trial of tremelimumab (CP-675,206) in patients with advanced refractory or relapsed melanoma. *Clinical Cancer Research*.

[56] Sangro B., Gomez-Martin C., de la Mata M. (2013). A clinical trial of CTLA-4 blockade with tremelimumab in patients with hepatocellular carcinoma and chronic hepatitis C. *Journal of Hepatology*.

[57] Ralph C., Elkord E., Burt D. J. (2010). Modulation of lymphocyte regulation for cancer therapy: a phase II trial of tremelimumab in advanced gastric and esophageal adenocarcinoma. *Clinical Cancer Research*.

[58] Barber D. L., Wherry E. J., Masopust D. (2006). Restoring function in exhausted CD8 T cells during chronic viral infection. *Nature*.

[59] Francisco L. M., Salinas V. H., Brown K. E. (2009). PD-L1 regulates the development, maintenance, and function of induced regulatory T cells. *The Journal of Experimental Medicine*.

[60] Okazaki T., Tanaka Y., Nishio R. (2003). Autoantibodies against cardiac troponin I are responsible for dilated cardiomyopathy in PD-1-deficient mice. *Nature Medicine*.

[61] Zou W., Chen L. (2008). Inhibitory B7-family molecules in the tumour microenvironment. *Nature Reviews Immunology*.

[62] Topalian S. L., Drake C. G., Pardoll D. M. (2012). Targeting the PD-1/B7-H1(PD-L1) pathway to activate anti-tumor immunity. *Current Opinion in Immunology*.

[63] Topalian S. L., Hodi F. S., Brahmer J. R. (2012). Safety, activity, and immune correlates of anti-PD-1 antibody in cancer. *The New England Journal of Medicine*.

[64] Brahmer J. R., Drake C. G., Wollner I. (2010). Phase I study of single-agent anti-programmed death-1 (MDX-1106) in refractory solid tumors: safety, clinical activity, pharmacodynamics, and immunologic correlates. *Journal of Clinical Oncology*.

[65] Lipson E. J., Sharfman W. H., Drake C. G. (2013). Durable cancer regression off-treatment and effective reinduction therapy with an anti-PD-1 antibody. *Clinical Cancer Research*.

[66] Gettinger S. N., Shepherd F. A., Antonia S. J. First-line nivolumab (anti-PD-1; BMS-936558, ONO-4538) monotherapy in advanced NSCLC: Safety, efficacy, and correlation of outcomes with PD-L1 status.

[67] Hardy B., Indjiia L., Rodionov G., Raiter A., Inbal A. (2001). Treatment with BAT monoclonal antibody decreases tumor burden in a murine model of leukemia/lymphoma. *International Journal of Oncology*.

[68] Hardy B., Niv Y., Fadaeev L., Raiter A. (2005). BAT mAb induces lymphopoiesis in nude mice. *International Immunology*.

[69] Berger R., Rotem-Yehudar R., Slama G. (2008). Phase i safety and pharmacokinetic study of CT-011, a humanized antibody interacting with PD-1, in patients with advanced hematologic malignancies. *Clinical Cancer Research*.

[70] Armand P., Nagler A., Weller E. A. (2013). Disabling immune tolerance by programmed death-1 blockade with pidilizumab after autologous hematopoietic stem-cell transplantation for diffuse large B-cell lymphoma: results of an international phase II trial. *Journal of Clinical Oncology*.

[71] Westin J. R., Chu F., Zhang M. (2014). Safety and activity of PD1 blockade by pidilizumab in combination with rituximab in patients with relapsed follicular lymphoma: a single group, open-label, phase 2 trial. *The Lancet Oncology*.

[72] Patnaik A., Kang S. P., Tolcher A. W. Phase I study of MK-3475 (anti-PD-1 monoclonal antibody) in patients with advanced solid tumors.

[73] Dong H., Zhu G., Tamada K., Flies D. B., van Deursen J. M. A., Chen L. (2004). B7-H1 determines accumulation and deletion of intrahepatic CD8^+^ T lymphocytes. *Immunity*.

[74] Latchman Y. E., Liang S. C., Wu Y. (2004). PD-L1-deficient mice show that PD-L1 on T cells, antigen-presenting cells, and host tissues negatively regulates T cells. *Proceedings of the National Academy of Sciences of the United States of America*.

[75] Butte M. J., Keir M. E., Phamduy T. B., Sharpe A. H., Freeman G. J. (2007). Programmed death-1 ligand 1 interacts specifically with the B7-1 costimulatory molecule to inhibit T cell responses. *Immunity*.

[76] Park J.-J., Omiya R., Matsumura Y. (2010). B7-H1/CD80 interaction is required for the induction and maintenance of peripheral T-cell tolerance. *Blood*.

[77] Chen D. S., Irving B. A., Hodi F. S. (2012). Molecular pathways: next-generation immunotherapy-inhibiting programmed death-ligand 1 and programmed death-1. *Clinical Cancer Research*.

[78] Furness A. J., Vargas F. A., Peggs K. S., Quezada S. A. (2014). Impact of tumour microenvironment and Fc receptors on the activity of immunomodulatory antibodies. *Trends in Immunology*.

[79] Herbst R. S., Gordon M. S., Fine G. D. (2013). A study of MPDL3280A, an engineered PD-L1 antibody in patients with locally advanced or metastatic tumors. *ASCO Meeting Abstracts*.

[80] Wolchok J. D., Kluger H., Callahan M. K. (2013). Nivolumab plus Ipilimumab in advanced melanoma. *The New England Journal of Medicine*.

[81] Sznol M., Kluger H. M., Callahan M. K. Survival, response duration, and activity by BRAF mutation (MT) status of nivolumab (NIVO, anti-PD-1, BMS-936558, ONO-4538) and ipilimumab (IPI) concurrent therapy in advanced melanoma (MEL).

[82] Meyer C., Cagnon L., Costa-Nunes C. M. (2014). Frequencies of circulating MDSC correlate with clinical outcome of melanoma patients treated with ipilimumab. *Cancer Immunology, Immunotherapy*.

[83] Kitano S., Postow M. A., Ziegler C. G. (2014). Computational algorithm-driven evaluation of monocytic myeloid-derived suppressor cell frequency for prediction of clinical outcomes. *Cancer Immunology Research*.

[84] Snyder A., Makarov V., Merghoub T. (2014). Genetic basis for clinical response to CTLA-4 blockade in melanoma. *The New England Journal of Medicine*.

[85] Hamid O., Schmidt H., Nissan A. (2011). A prospective phase II trial exploring the association between tumor microenvironment biomarkers and clinical activity of ipilimumab in advanced melanoma. *Journal of Translational Medicine*.

[86] Ji R.-R., Chasalow S. D., Wang L. (2012). An immune-active tumor microenvironment favors clinical response to ipilimumab. *Cancer Immunology, Immunotherapy*.

[87] Wilgenhof S., Four S. D., Vandenbroucke F. (2013). Single-center experience with ipilimumab in an expanded access program for patients with pretreated advanced melanoma. *Journal of Immunotherapy*.

[88] Delyon J., Mateus C., Lefeuvre D. (2013). Experience in daily practice with ipilimumab for thetreatment of patients with metastatic melanoma: anearly increase in lymphocyte and eosinophil countsis associated with improved survival. *Annals of Oncology*.

[89] Kelderman S., Heemskerk B., van Tinteren H. (2014). Lactate dehydrogenase as a selection criterion for ipilimumab treatment in metastatic melanoma. *Cancer Immunology, Immunotherapy*.

